# Molecular changes associated with increased TNF-α-induced apoptotis in naïve (T_N_) and central memory (T_CM_) CD8+ T cells in aged humans

**DOI:** 10.1186/s12979-017-0109-0

**Published:** 2018-01-19

**Authors:** Sudhir Gupta, Houfen Su, Sudhanshu Agrawal, Sastry Gollapudi

**Affiliations:** 1 Program in Primary Immunodeficiency and Aging, Division of Basic and Clinical Immunology, University of California, Irvine, USA; 2 Division of Basic and Clinical Immunology, Medical Sci. I, C-240, University of California at Irvine, Irvine, CA 92697 USA

**Keywords:** TNF-α, A20, TRAF-2, RIP, cFLIP, NF-κb

## Abstract

**Background:**

Progressive T cell decline in aged humans is associated with a deficiency of naïve (T_N_) and central memory (T_CM_) T cells. We have previously reported increased Tumor necrosis factor-α (TNF-α)-induced apoptosis in T_N_ and T_CM_ T cells in aged humans; however, the molecular basis of increased apoptosis remains to be defined. Since expression of TNF receptors (TNFRs) was reported to be comparable in young and aged, we investigated signaling events downstream of TNFRs to understand the molecular basis of increased TNF-α-induced apoptosis in aged T_N_ and T_CM_ CD8+ cells.

**Results:**

The expression of TRAF-2 and RIP, phosphorylation of JNK, IKKα/β, and IκBα, and activation of NF-κB activation were significantly decreased in T_N_ and T_CM_ CD8+ cells from aged subjects as compared to young controls. Furthermore, expression of A20, Bcl-x_L_, cIAP1, and FLIP-_L_ and FLIP-_S_ was significantly decreased in T_N_ and T_CM_ CD8+ cells from aged subjects.

**Conclusions:**

These data demonstrate that an impaired expression/function of molecules downstream TNFR signaling pathway that confer survival signals contribute to increased apoptosis of T_N_ and T_CM_ CD8+ cells in aged humans.

## Background

Aging is associated with a progressive decline in immune responses including impaired proliferative and effector responses, impaired T cell signaling, and increased frequency of infections [1–12]. However, molecular mechanisms for immune dysfunction with age are poorly understood. Following antigenic stimulation naïve CD8+ T cells (T_N_) undergo activation and clonal expansion to generate effector CD8+ T cells. After clearance of antigen, majority of effector cells undergo apoptosis, and a subpopulation of effectors cells is retained as long-term memory cells [13]. Based upon their homing properties, and expression of adhesion molecules and chemokine receptors, memory T cells are classified into central memory (T_CM_) and effector memory (T_EM_) CD8+ T cells [14–22]. We, and others have reported their characteristics with regard to proliferative response, cytokine production, effector properties, and sensitivity to apoptosis via death receptors, mitochondrial, and endoplasmic reticulum stress signaling pathways [21, 23–25].

TNF-α is a pleiotropic cytokine that activates T cells via both TNF-RI and TNF-RII and mediates both apoptotic and survival signals [26–35]. TNFα-mediates its biological functions predominantly via TNFR-I. Following binding of TNF-α to TNFR-I, the TNFR-associated death domain (TRADD) is recruited to TNFR-I forming a platform for downstream signaling. TNRF-associated factor 2 (TRAF2) and receptor-interacting protein kinase 1 (RIPK1) are recruited to TRADD forming a signaling complex. TRADD also recruits fas-associated death domain (FADD), which initiates activation of apical caspases resulting in activation of effector caspases, and apoptosis. Both RIPK1 and TRAF2 recruit IKKα and IKKβ to the signaling complex resulting in NF-κB activation [36, 37]. NF-κB translocates to the nucleus, binds to the promoter, and induces a number of anti-apoptotic genes, including FLIP, IAPs, A20, Bcl-x_L_ [32–34]. TRAF2 also activates MAP kinase/JNK pathway; prolonged JNK activation may result in apoptosis [38].

In human aging, TNF-α production is increased [9–12]. A number of investigators have reported increased sensitivity of T cells, CD4+ and CD8+ T cells and their subsets to death receptors (CD95 and TNF-) mediated apoptosis [25, 39–41]. In aging humans, there is a deficiency in T_N_, which in part appears to be associated with increased sensitivity to death-receptor-induced apoptosis [42–47]. In addition, we have reported a deficiency of T_CM_ CD8+ T cells in aging [22]. Furthermore, we have reported that the expression of TNF receptors is comparable between young and aged subjects [23, 48], therefore suggesting that mechanism(s) for increased sensitivity of T_N_ and T_CM_ CD8+ cells to apoptosis in aging must lie in signaling pathway downstream of TNFRs. In contrast, effector memory CD8+ T cells (T_EM_ and T_EMRA_) are resistant to apoptosis, and there is no significant different in TNF-α-induced apoptosis in these subsets between young and aged subjects [48].

In this study we present molecular mechanisms of increased sensitivity of purified T_N_ and T_CM_ CD8+ T in aged humans to TNF-α-induced apoptosis by investigating signaling downstream of TNFRs. Our data show that increased apoptosis in T_N_ and T_CM_ CD8+ cells from aged subjects is due to decreased expression/function of molecules involved in the signaling pathway involved in cell survival.

## Methods

### Subjects

Peripheral blood was obtained from 15 healthy young (age 21–35 years with a mean age of 34 years; 9 female and 6 male) and 15 aged (age 65–88 years with a mean age of 72 years, 9 female and 6 male) subjects. Aging subjects belong to middle-class social status and living independently in senior community of Laguna Woods, California. Aging subjects were required to discontinue any and all nutritional supplements at least one week prior to blood draw, to avoid any effect of anti-oxidants, which are commonly used by aging population.

### Reagents and monoclonal antibodies

Directly conjugated monoclonal antibodies against CD8 and CD45RA and their isotypes and unconjugated CD8 antibodies were obtained from BD Biosciences (San Diego, CA). Anti-CCR7 and isotypes were purchased from R & D systems, Minneapolis, MN, and anti-CD3/CD28 was Life Technology, Camarillo, CA. TNF-α was obtained from Laguna Scientific, Laguna Niguel, CA. Antibodies to FLIP and IAP were purchased from Transduction Laboratories, San Diego, CA, and antibodies to phospho IKKα/β, phospho IκB, phospho JNK, phosphor TAK1 TAK1, and TAB2 were purchased from Cell Signaling Technologies, Inc. Beverly, MA. Antibodies to A20, TRAF2 and RIPK1 were obtained from Santa Cruz Biotechnology, Dallas, TX. In Situ Cell Death Detection Kit was purchased from Boehringer-Manheim, Indianapolis, IN.

### Isolation of T_N_ and T_CM_ CD8+ T cells and culture conditions

Purified T_N_ and T_CM_ CD8+ T were separated from healthy young and aged subjects to determine age-related changes rather than simple differences between young and aged subjects. Peripheral blood mononuclear cells (MNCs) were activated with anti-CD3/CD28 monoclonal for 48 h. Cells are washed and used for purification of T_N_ and T_CM_ CD8+ T cells (cells are activated because freshly isolated human T cells are resistant to all types of death receptor induced apoptosis). First, CD8^+^ T cells were isolated by negative selection with EasySep CD8+ enrichment cocktail and magnetic nanoparticles (Stem cell Technologies, Vancouver, BC, Canada). Briefly, unwanted cells were specifically-labelled with bispecific tetrameric antibody complexes that recognize unwanted cells and dextran. Dextran-coated magnetic nanoparticles were added and magnetically labeled cells were then separated from unlabeled target cells (CD8+ T cells) using a magnet. Cells obtained are more than 98% CD8 ^+^. T_N_ (CD8+, CD45RA+ CCR7+) and T_CM_ T-cells (CD8 + CD45RA- CCR7+) were purified to more than 95% by a two-step procedure. First, CD8+ T cells are separated into CD45RA+ and CD45RA- subpopulations by anti-CD45 RA antibody coated Petri dishes. In the second step, CCR7+ T cells are isolated by positive selection using EasySep PE selection kit (Stem cell Technologies). Briefly, CD45RA+ and CD45RA- T cells are labeled with phycoerythrin (PE)-conjugated anti-CCR7 antibody. The labeled cells are then incubated with bispecific tetrameric antibody complexes that recognize PE labeled cells and dextran. After 15 min incubation at room temperature, dextran-coated magnetic nanoparticles are added and magnetically labeled cells are separated from unlabeled cells using a magnet. Positively enriched cells are labeled with APC conjugated anti-CD45 and PerCp-conjugated anti-CD8 and the purity of isolated populations are determined by multicolor analysis using FACSCalibur. Purified T_N_ and T_CM_ CD8+ T cells were activated with TNF-α to study phosphorylation of signaling molecules by Western blotting.

TNF-α-induced apoptosis was assayed in activated T_N_ and T_CM_ CD8+ because ex-vivo freshly isolated T cell subsets are resistant to TNF-α-induced apoptosis. Furthermore, phenotypes of T_N_ cells and T_CM_ were largely maintained following 48 h of anti-CD3/CD28 stimulation of MNCs.

### Apoptosis

Purified T_N_ and T_CM_ CD8+ T cells were stimulated with TNF-α for 48 h to assay for apoptosis. Apoptosis was measured by TUNEL assay (terminal deoxyribonucleotidyl transferase (TDT)-mediated dUTP- nick end labeling). Briefly, TNF-activated purified T_N_ and T_CM_ CD8+ cells were fixed with 2% formaldehyde for 30 min at room temperature, washed with phosphate buffer saline (PBS), and permeabilized with sodium citrate buffer containing 0.1% Triton X-100 for 2 min on ice. Following washing, cells were incubated with FITC-conjugated dUTP in the presence of TdT enzyme solution containing 1 M potassium cacodylate and 125 mM Tris-Hcl, Ph 6.6 for an hour at 37 °C. Following incubation, cells were washed with PBS, and 10,000 cells were acquired and analyzed by multicolor flow cytometry using FACSCalibur.

### Flow cytometry

MNCs activated with anti-CD3/CD28 for 48 h and, then exposed to TNF-α for 10 min. Cell were first surface stained by CCR7 FITC, CD45RA APC, CD8PerCP antibodies and isotype controls. Stained cells were then fixed by 2% paraformaldehyde for 10 min at room temperature, washed and permeabilized by 90% methanol for 15 min on ice. Cells were washed and kept in PBS/2% FBS for 60 min for rehydration and then stained with purified antibodies to cIAP1and A20 and isotype controls. Cells were washed and stained with secondary PE conjugated goat anti- rabbit antibody.. First cells were gated for CD8+ T cells, and then gated for T_N_ (CD8+, CD45RA+ CCR7+) and T_CM_ T-cells (CD8 + CD45RA- CCR7+) cells. These gated cells were then analyzed for the expression of cIAP1 and A20. Ten thousand sells were acquired, and were enumerated using FACSCalibur. Data were analyzed by Flow jo software.

### Western blotting

Purified T_N_ and T_CM_ cells activated with TNF-α were lysed with lysis buffer (Cell Signaling). Aliquots of cell lysates containing 50μg of total protein were resolved by SDS-PAGE and transferred onto membranes (Millipore, Bedford, MA) by electro blotting. The membranes were blocked for 1 h at room temperature in TBS-T buffer with 5% nonfat dried milk and incubated with 1μg/ml primary antibodies listed above in reagents and anti-β actin antibody as loading control used dilution 1:5000 overnight at 4C. The blots were washed three times for 20 min with TBS-T buffer and then incubated with HRP-conjugated secondary antibodies (1:5000–1:10,000 dilution) for 1 h at room temperature. After washing three times for 20 min in TBS-T buffer, blots were developed using enhanced chemiluminescence reagents (ECL, Thermo Scientific Pierce Biotech, Rockford, IL) and exposed to Clear Blue X-Ray Film. Blots were scanned with densitometer.

#### ELISA for NF-κB activity

DNA-binding activity of NF-κB was measured using an ELISA kit for NF-κB p65 according to manufacturer’s protocol (Active Motif, San Diego, CA). The 96-well plates were coated with the oligonucleotide specific for NF-κB binding and the bound NF-κB was measured using anti-NF-κB p65 antibody as described (23). This method provides advantage over traditional EMSA assay in that it is a sensitive assay without using radioactivity, and a large number of samples with smaller number of cells can be analyzed simultaneously.

Statistical analysis was performed by student t test.

## Results

### Increased sensitivity to TNF-α-induced apoptosis in T_N_ and T_CM_ CD8 cells in aged subjects

**Fig. 1:**
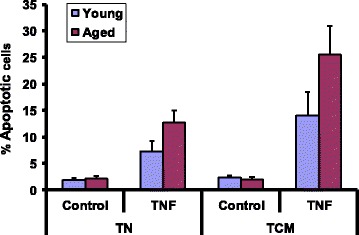
TNF-α-induced apoptosis in T_N_ and T_CM_ CD8+ T cells. Activated purified T_N_ and T_CM_ CD8+ T cells subsets were incubated in the absence or presence of TNF-α for 48 h and apoptosis was measured by TUNEL assay using FACSCalibur. Ten thousand cells were acquired. Data for spontaneous and TNF-α-induced apoptosis from 10 young and 10 aged subjects are presented as percent of TUNEL positive cells. A significantly increased (*P* < 0.001) apoptosis was observed in aged subjects. No significant difference was observed in spontaneous apoptosis between young and aged subjects

Purified activated T_N_ and T_CM_ CD8+ cells from young and aged subjects were incubated in the absence or presence of TNF-α for 48 h. Apoptosis was measured by TUNEL assay. Fig. 1 shows data from 10 young and 10 aged subjects. No significant difference was observed in spontaneous apoptosis between young and aged group. However, a significantly higher (*P* < 0.001) TNF-α-induced apoptosis was observed in both T_N_ and T_CM_ CD8+ T cells from aged as compared to young controls. This is in agreement with previous reports [23, 48].

### TRAF-2 and RIP expression is decreased in aged T_N_ and T_CM_

**Fig. 2:**
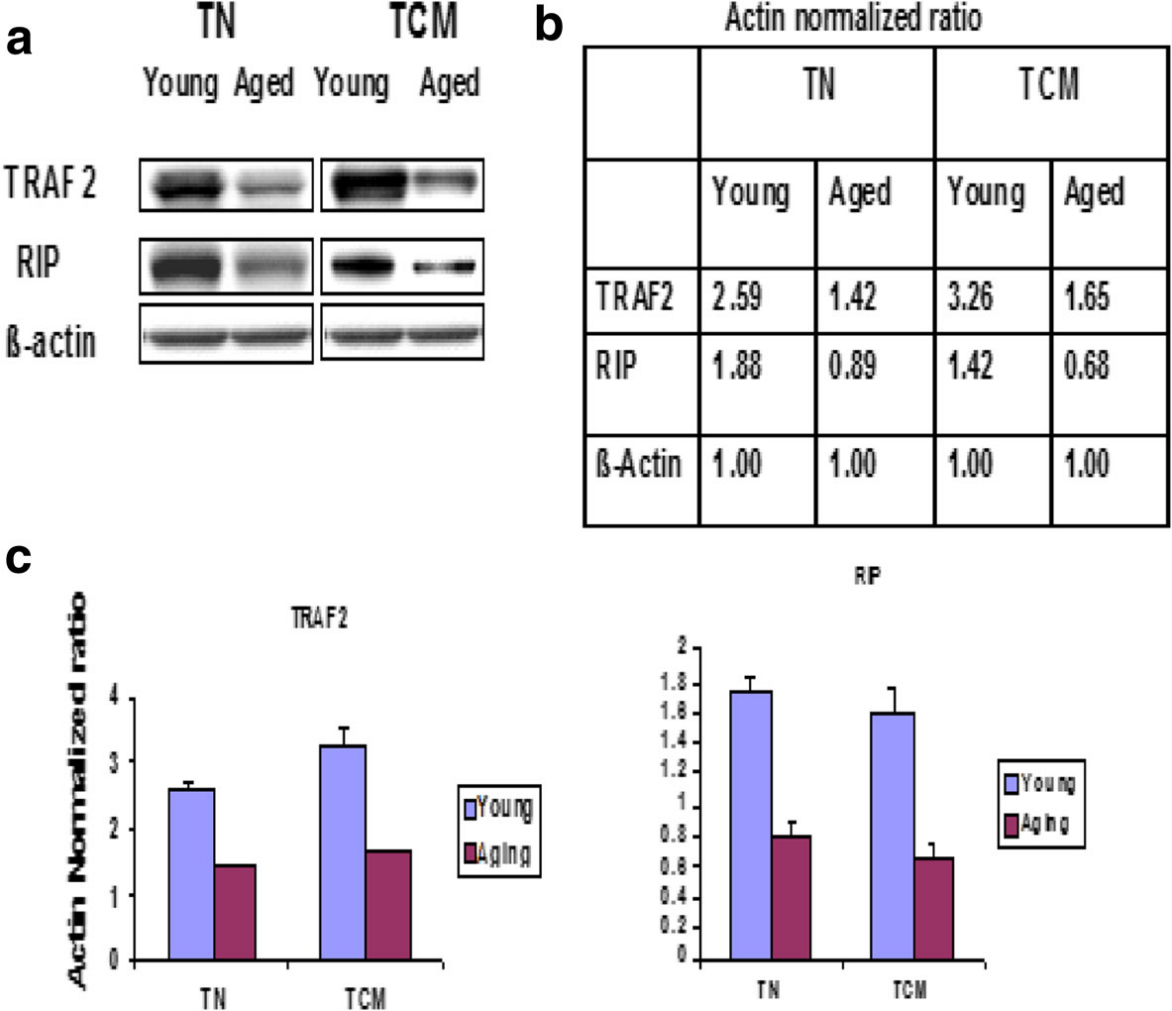
TRAF-2 and RIP expression in T_N_ and T_CM_ CD8+ T cells. Protein extracted from purified T_N_ and T_CM_ CD8+ T cells from aged and young subjects was analyzed by Western blotting using specific antibodies. [**a**] Western blots from a representative experiment, [**b**] densitometry data, and [**c**] cumulative densitometry data from five each young and aged subject. Both TRAF-2 and RPI expression was significantly increased (*P* < 0.001) in aged subjects

Since TNFRI and TNFRII expression on aged T_N_ and T_CM_ CD8+ cells is comparable to young subjects [23, 48] we reasoned that an impaired expression/function of adapter molecule TRAF2 may play an important role in increased sensitivity of T_N_ and T_CM_ CD8+ T cells in aged humans, via decreased activation of RIP and TAK1 resulting in decreased NF-κB activity and an impaired induction of NF-κB target anti-apoptotic genes. Therefore, first we examined the expression of TRAF-2 and RIPK1 in T_N_ and T_CM_ CD8+ T cells. Proteins were extracted from purified subsets from young and aged subjects and the expression of these molecules was analyzed by Western blotting with specific antibodies and analyzed by densitometry. Actin was used as a loading internal control. Fig. 2a shows a representative Western blots and Fig. 2b shows data from densitometry of Western blot normalized for actin loading control. Figures 2c shows cumulative densitometry data of Western blot of T_N_ and T_CM_ CD8+ T cells from five young and five aged subjects normalized for actin loading control. The expression of TRAF-2 and RIPK1 was significantly decreased (*P* < 0.001) in T_N_ and T_CM_ CD8+ T cells from aged subjects.

### Phosphorylated TAK1 is decreased in aged T_N_ and T_CM_ CD8 cells

**Fig. 3:**
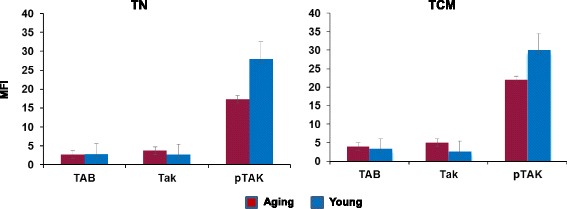
Expression of TAB2, TAK1 and phospo TAK1. Purified T_N_ and T_CM_ CD8 + T cells were incubated in the absence or presence of TNF-α for 10 min, and the expression of TAB2 and TAK1, and phosphorylation of TAK-1 were measured with specific antibodies and flow cytometry. Isotype antibodies were used as background control. Data were analyzed for fluorescence intensity (MFI) as an indicator of density of molecules. Cumulative data from 5 young and 5 aged subjects show a significantly decreased (*P* < 0.004) pTAK1 in aged subjects as compared to young subjects

RIPK1 activates TAK-1 via recruitment of TAK-1 complex and interaction of K^63^ubiquitin chains to TAB2 and subsequent phosphorylation of TAK1 [49]. Since RIPK1 levels are decreased in T_N_ and T_CM_ CD8+ T cells in aged, we examine the expression of TAB2 and TNF-α-induced phosphorylation of TAK-1 in T_N_ and T_CM_ CD8+ T cells. Purified T_N_ and T_CM_ CD8 + T cells were incubated in the presence or absence of TNF-α, and the expression of TAB2 and TAK1, and phosphorylation of TAK-1 was measured with specific antibodies and flow cytometry. Isotype antibodies were used as background control. Figure 3 represents cumulative data of mean fluorescence intensity (density of molecules) from five each young and aged subjects. TAB2 and TAK1 expression was comparable; however, phosphorylated TAK-1 was significantly decreased (*P* < 0.004) in aged cells.

### TNF-α-induced phosphorylation of IKKα/β, IκBαand activation of NF-kB is impaired in aged T_N_ + T_CM_ CD8 cells

TAK1 phosphorylates IKKβ, which in turn phosphorylates IκBα, resulting in the release and activation of NF-κB (p65/p50), providing a survival signal [50]. In contrast, TAK1 activates JNK that promotes apoptosis [51]. Therefore, we examined TNF-α-induced phosphorylation of JNK, IKKα/β, IκBα, and activation of NF-κB in young and aged subjects.

**Fig. 4:**
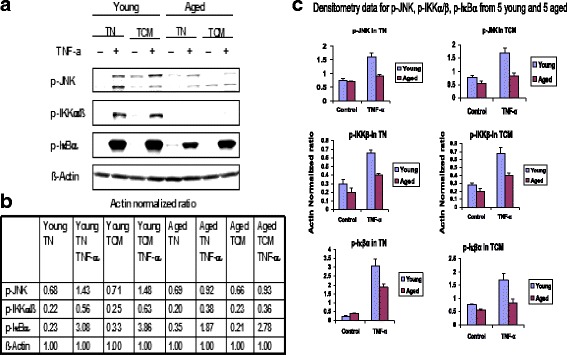
Effect of TNF-α on the phosphorylation of JNK, IKKβ, IκB in T_N_ and T_CM_ CD8+ T cells. Purified T_N_ and T_CM_ CD8+ T cells from young and aged subjects were stimulated with TNF-α for 10 min and then analyzed for expression of phospho JNK, IKKβ, IκB, using specific antibodies. β-actin was used as a loading control. [**a**] Western blot from one such experiment, [**b**] densitometry data, and [**c**] shows densitometry data from 5 each young and aged subjects. A significant decrease in pJNK, pIKKβ, and pIκB (*P* < 0.05, *P* < 0.01) was observed in aged subjects as compared to young subjects

Purified T_N_ and T_CM_ CD8+ T cells were activated with TNF-α for 10 min, and the protein was extracted and analyzed by Western blotting, using specific antibodies against phospho IKKα/β, phospho IκBα, and phospho JNK. A representative Western blot is shown in Fig. 4a and the densitometry data from these blots normalized for actin loading control are shown in Fig. 4b. Fig. 4c shows cumulative densitometry data of Western blots from five aged subjects and five young subjects. The levels of phospho JNK, IKKα/β, and IκBα in T_N_ and T_CM_ CD8+ T cells from aged subjects were significantly decreased (*P* < 0.05- < 0.01) as compared to young subjects. No difference was observed in the expression of NEMO (data not shown). While JNK signaling can contribute to TNF-induced apoptosis, it is unlikely that decreased JNK activation contributes to increased apoptosis in aged subjects under these experimental conditions.

**Fig. 5:**
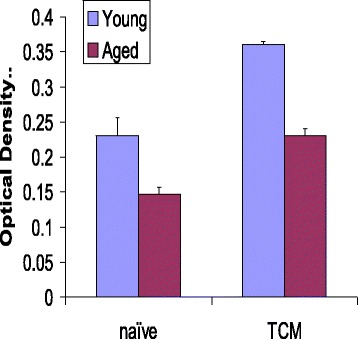
TNF-α-induced NF-κB activity in T_N_ and T_CM_ CD8+ T cells by ELISA-based DNA binding assay. Purified T_N_ and T_CM_ CD8+ T cell subsets were activated with TNF-α for 10 min and NF-κB activity was measured by ELISA-based DNA binding activity. Data are shown from five young and five aged subjects. A significantly lower (*P* < 0.01) NF-κB activation was observed in aged subjects

We also compared NF-κB activity in T_N_ and T_CM_ CD8+ T cells in young and aged subjects. Purified subsets were activated with TNF-α for 10 min, and NF-κB activity was measured by ELISA-based DNA binding activity. Data from five young and five aged subjects are shown in Fig. 5. Both T_N_ and T_CM_ CD8+ T cells from aged subjects show significantly lower NF-κB activity following TNF-α activation (*P* < 0.01) as compared to young subjects.

### Bcl-X_L_, FLIP_L_, FLIP_S_, A20 and cIAP expression is decreased in T_N_ + T_CM_ CD8 cells from aged humans

**Fig. 6:**
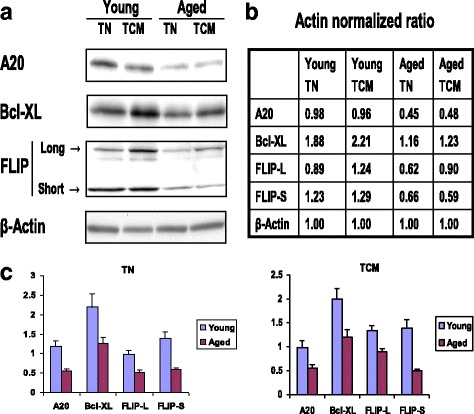
Expression of A20, Bcl-X_L_, and FLIP_L_ and FLIPs in T_N_ and T_CM_ CD8+ T cells. Protein extracted from purified T_N_ and T_CM_ CD8+ T cells from aged and young subjects was analyzed by Western blotting using specific antibodies. [**a**] shows a representative Western blot for A20, Bcl-X_L_, and FLIP expression in T_N_ and T_CM_ CD8+ T cells [**b**] shows densitometry data from these blots, [**c**] shows cumulative densitometry data (mean ± sd) of Western blots from five young and five aged subjects. T_N_ and T_CM_ CD8+ T cells subsets from aged subjects display significantly decreased expression of A20 (P < 0.05), Bcl-X_L_, (*P* < 0.01), FLIP_L_ (*P* < 0.05) and FLIPs (*P* < 0.05)

Since NF-κB activates a number of anti-apoptotic genes [52–60], next we examined the expression of cIAP, A20, FLIP and Bcl-x_L_ in purified T_N_ and T_CM_ CD8+ T cells in aged and young subjects. Protein extracted from purified T_N_ and T_CM_ CD8+ T cells from aged and young subjects was analyzed by Western blotting using specific antibodies. A representative Western blot for A20, Bcl-X_L_, and FLIP_L_, FLIP_S_ expression in T_N_ and T_CM_ CD8+ T cells is shown in Fig. 6a and densitometry data from these blots are shown in Fig. 6b. Fig. 6c shows cumulative densitometry data (mean ± sd) of Western blots from five young and five aged subjects. The expression of A20, FLIP_L_ and FLIP_S_, and Bcl-x_L_ was significantly (*P* < 0.05- < 0.001) decreased in aged subjects.

**Fig. 7:**
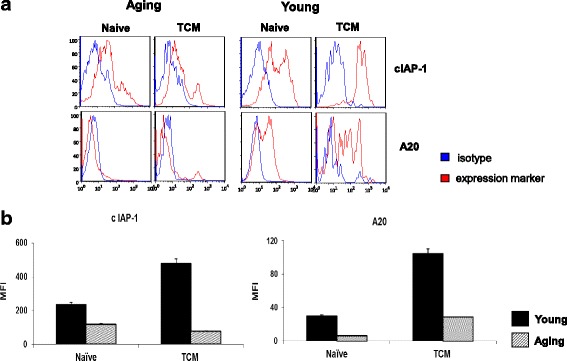
Expression of A20 and cIAP1 by flow cytometry. MNCs activated with anti-CD3/CD28 for 48 h and, then exposed to TNF-α for 10 min. Cell were first surface stained by CCR7 FITC, CD45RA APC, CD8PerCP antibodies and isotype controls. Stained cells were then fixed and permeabilized, and then stained with purified antibodies to cIAP1and A20 and isotype controls. Cells were washed and incubated with secondary PE conjugated goat anti- rabbit antibody. First cells were gated for CD8+ T cells, and then gated for T_N_ (CD8+, CD45RA+ CCR7+) and T_CM_ T-cells (CD8 + CD45RA- CCR7+) cells. These gated cells were then analyzed for the expression of cIAP1 and A20. **a** is a representative FACS plot. Blue line represents isotype control, and red line is for A20 and cIAP1. **b** shows cumulative data for MFI from 5 young and 5 aged subjects. T_N_ and T_CM_ CD8+ T cells from aged subjects show significantly decreased (*P* < 0.001) expression of both A20 and cIAP

Since antibodies to A20 and cIAP for flow cytometry became available, and flow cytometry is experimentally less cumbersome and less time consuming than Western blotting, we analyzed the expression of A20 and cIAP in T_N_ and T_CM_ CD8+ T cells from aged and young subjects by flow cytometry. Data in Fig. 7a is a representative FACS plots, and data in Fig. 7b is cumulative from 4 young and 4 aged subjects (mean ± sd). The expression of A20 (similar to Western blot data in Figure 6) and cIAP1 is significantly decreased (*P* < 0.001) in T_N_ and T_CM_ CD8+ T cells from aging as compared to young subjects. These data show that flow cytometry for the analysis of these molecules is a reliable technique, and has advantage over Western blotting in that [a] analysis can be performed on small number of cells, [b] there is no requirement of purification of T_N_ and T_CM_ CD8+ T cells, and [c] provide better quantitative analysis.

## Discussion

Following virus infection or antigen stimulation, naïve T cells undergo a series of proliferative and differentiation steps resulting in the development of effector and memory cells [3]. The differential expression of adhesion molecule (CD62L) and chemokine receptor (CCR7) on memory T cells results in their homing either to lymph nodes (T_CM_) or to extra nodal sites such as liver and lung (T_EM_) [14–21]. Both our group, and others have reported decreased in T_N_ and T_CM_ T cells in aged humans [22, 42–47]. Although a role of thymus in decreased out put of naïve T cells is well-established, we and others have also shown that an increased apoptosis may also contributes to decreased T_N_ cells in aging [42, 47]. Previously we have reported that T_N_ and T_CM_ CD8+ T cells are more sensitive to both TNF-α-and CD95-induced apoptosis via activation of caspases as compared to T_EM_ and T_EMRA_ CD8+ T cells [23, 42, 48]; however, expression of TNFRs is comparable to young subjects [23, 48]. Our present data also show increased TNF-α-induced apoptosis in both T_N_ and T_CM_ CD8+ from aged subjects, which may contribute to their deficiency in aged humans.

TNF-α is a proinflammatory molecule that plays an important role in diverse cellular events including induction of cytokines, cellular proliferation, differentiation, survival and apoptosis [26, 35]. TNF-α-mediates these processes via TNFR-I and/or TNFR-II, apoptosis is predominantly mediated via TNFR-I.

Previously, we have shown that expression of TNFR-I and TNFR-II is comparable in all four subsets of CD8+ T cells (T_N_, T_CM_, T_EM_, T_EMRA_); however, T_EM_ and T_EMRA_ CD8+ T cells are resistant to TNF-α-induced apoptosis [48]. Furthermore, in aging, apoptosis and activation of caspase 3 and caspase 8 are increased only in T_N_ and T_CM_ CD8+ T cells [48]. Therefore, these data suggest that the differences in TNF-α-induced apoptosis in aged T_N_ and T_CM_ are due to differences in signaling pathway downstream of TNFRs.

The interaction and binding of TNF-α to TNFR-I leads to trimerization of TNFR-I and via death domain and by protein-protein interaction recruits TRADD, which acts as a platform to recruit other proteins including FADD, TRAF2, and RIPK1, forming a signaling complex that activates NF-κB, which induces anti-apoptotic genes. We have shown that deficiency of FADD plays an important role in an increased apoptosis of lymphocytes from aged humans [61]. FADD expression is increased in lymphocytes from aged subjects, and transfection of aged lymphocytes with FADD dominant negative plasmid significantly reduced TNF-induced apoptosis in aged lymphocytes comparable to young subjects. Furthermore, we demonstrated that an overexpression of FADD in lymphocytes from young subjects with wild-type FADD resulted in an increased apoptosis of young lymphocytes to a level similar to aged subjects.

RIPK1, a multifunctional protein, and TRAF-2 are required for the activation of NF-κB. It has been demonstrated that in TNF-induced apoptosis caspase-8 cleaves RIPK1 [62]. TRAF2 together with ubiquitin conjugating enzyme complex catalyzes the synthesis of a unique polyubiquitin chain K^63^ of ubiquitin [63–66]. K^63^ polyubiquitination of RIPK1 leads to its activation and recruitment of TAK1 complex and IKK complex [50, 67–70]. This results in the activation of TAK1 kinase complex through interaction between the K^63^ polyubiquitin chain and an ubiquitin-binding domain on TAB2 regulatory units of TAK1 complex [50] and of IKKγ (NEMO) via interaction with K^63^ polyubiquitin chain [69]. TAK1 phosphorylates and activate IKKβ, resulting in phosphorylation and degradation of IκBα, and activation of NF-κB activation [50, 71]. In the current study, we observed decreased expression of both TRAF2 and RIP. TAK1 and not TAB1 or TAB2 plays a role in multiple signaling pathways [72]. In this study we did not see any difference in TAB2 expression in T_N_ and T_CM_ CD8 cells between young and aged; however, we observed decreased phosphorylation of TAK1, IKKβ, and IκBα, and decreased activation of NF-κB in T_N_ and T_CM_ CD8 cells. Taken together signaling molecules downstream of TNFR appear to be responsible for increased sensitivity to TNF-α-induced apoptosis in T_N_ and T_CM_ CD8 cells from aged humans.

The anti-apoptotic genes that are target of NF-κB activation include *cIAP1, cIAP2, Bcl-x*_*L*_*, A20 and FLIP* show decreased expression in aged naïve and T_CM_ CD8+ T cells [52–60].

A20 (tumor necrosis factor alpha-induced protein 3), a ring finger ubiquitin-modifying enzyme, is essential for the termination of TNF-α-induced activation of NF-kB and inhibition of TNF-induced apoptosis [56–58]. A20 has dual activity in that it inhibits apoptosis as well as activates NF-κB [56, 73]**.** Interaction of A20 and cIAP with TRAF2 results in the releases of cIAP from the TRAF2-signaling complex, and allows these proteins to exert their anti-apoptotic effects. Our data show decreased expression of A20 and cIAP in aged T_N_ and T_CM_ CD8+, which are more sensitive to TNF-α-induced apoptosis as compared to young. Therefore, A20 deficiency in aging may be contributing to both increased apoptosis and inflammation. Our data suggest that in primary human CD8+ T cells A20 may function preferentially as an anti-apoptotic molecule.

IAP family proteins have a key role in the inhibition of apoptosis [55, 74, 75]. The cIAP-1 and cIAP2 are structurally homologous proteins. cIAP1 is recruited to DISC of TNFR-I by TRAF-2. Previously we have reported decreased expression of cIAP in CD4+ and CD8+ T cells in aging [76]. In this study we observed decreased expression of cIAP1 in aged T_N_ and T_CM_ CD8 cells as compared to young subjects, which may contribute to increased sensitivity to TNF-α-induced apoptosis in aged.

cFLIP, an apoptosis inhibiting molecules is a target of NF-κB [52]. FLIP comes in two alternatively spliced forms, the cFLIP_L_ and cFLIPs. cFLIPs contains two death effector domains (DED) and inhibits procaspase-8 activation, whereas, c-FLIP_L_ is enzymatically inactive. In addition to its inhibitory effect on procaspase-8 activation, cFLIP by associating with Raf-1activate MEK1, which subsequently activates ERK. cFLIP associates with TRAF2, resulting in NF-κB activation [53, 54, 77, 78]. cFLIP_L_ inhibits the interaction of caspase 8 prodomain with RIP1 death domain, and regulates caspase 8-dependent NF-κB activation [79]. Our data show a significant decreased expression of both cFLIP_L_ and cFLIP_S_ in T_N_ and T_CM_ CD8+ T cells in aged as compared to young subjects. It remains to determine whether decreased FLIP expression contribute to increased TNF-a-induced activation of caspase-8 and caspase-3 in T_N_ and T_CM_ CD8+ T cells in aged humans (48).

## Conclusions

Our data demonstrate that an impaired expression of adaptor proteins resulting in decreased activation of IKK pathway and decreased NK-κB activation, and decreased expression of anti-apoptotic molecules that are target of NF-κB might play a role in increased sensitivity of T_N_ and T_CM_ CD8+ T cells, thus contributing to their deficiency and T cell dysfunction in aged humans. However, data presented are correlative, and in vitro overexpression of these molecules may provide the mechanistic explanation for increased sensitivity of T_N_ and T_CM_ CD8+ T cells in aged humans.

## Abbreviations

c-FLIP: FLICE-like inhibitory protein
cIAP1: cellular inhibitor of apoptosis 1
FADD: fas-associated death domain
RIPK1: receptor-interacting protein kinase 1 (RIPK1)
T_CM_: Central memory T cells
T_N_: Naïve T cells
TNFR-1 and TNF-R-II: Tumor necrosis factor I and II
TRADD: TNFR-associated death domain
TRAF2: TNRF-associated factor 2
